# A Silver Nanoparticle-Modified Evanescent Field Optical Fiber Sensor for Methylene Blue Detection

**DOI:** 10.3390/s130303986

**Published:** 2013-03-21

**Authors:** Ji Luo, Jun Yao, Yonggang Lu, Wenying Ma, Xuye Zhuang

**Affiliations:** 1 State Key Laboratory of Optical Technologies on Nano-Fabrication and Mirco-Engineering, Institute of Optics and Electronics, Chinese Academy of Sciences, Chengdu 610209, China; 2 Lightweight Optics and Advanced Materials Technology Center, Chinese Academy of Sciences, Chengdu 610209, China; E-Mail: Chuck350@yahoo.cn; 3 Department of Communication Engineering, Chengdu University of Information Technology, Chengdu 610225, China; E-Mail: mwy@cuit.edu.cn; 4 East China Institute of Optoelectronic Integrated Devices, Bengbu 233042, China; E-Mail: zxye8888@hotmail.com

**Keywords:** evanescent field optical fiber sensors, silver nanoparticles, MEMS microchannel chip, sensitivity, methylene blue detection

## Abstract

A silver nanoparticle-modified evanescent field optical fiber sensor based on a MEMS microchannel chip has been successfully fabricated. Experimental results show that the sensor response decreases linearly with increasing concentration of analyte. Over a range of methylene blue concentrations from 0 to 0.4 μmol/mL, the sensor response is linear (R = 0.9496). A concentration variation of 0.1 μmol/mL can cause an absorbance change of 0.402 dB. Moreover, the optical responses of the same sensing fiber without decoration and modified with silver nanoparticles have also been compared. It can be observed that the output intensity of the Ag nanoparticle-modified sensor is enhanced and the sensitivity is higher. Meanwhile, the absorbance spectra are found to be more sensitive to concentration changes compared to the spectra of the peak wavelength.

## 1. Introduction

Optical fiber sensors have been studied now for over thirty years. Along with the development of various techniques and sensing measurements, an abundance of optical fiber sensors have been investigated and commercialized [1,2] and optical fiber biosensors for the diagnosis of diseases, food, and environmental detection of biological agents have been increasingly in demand over the past few decades. Traditionally, labeling of either the antibody or the receptor on the optical fiber surface for the detection is needed, however, in general the necessary labeling procedures are tedious and sometimes extra errors may be introduced. To avoid these problems, development of label-free optical fiber sensing is needed to satisfy the needs of emerging chemical and biological detection technologies.

The evanescent field optical sensor is an excellent promising candidate for label-free optical fiber sensing. Recently, evanescent field optical sensors have been used in a wide range of applications for their high accuracy, low cost, and rapid detection techniques, which are particularly useful for monitoring environmental samples and toxic substances [3]. The principle of the evanescent optical fiber is usually based on reflectance, absorbance, and fluorescence detection. Nevertheless, the development of the evanescent field optical fiber has stagnated because of the sensitivity limitation that does not satisfy the rigorous measurement demands of some analytes with fractional contents. As a consequence, efforts directed at improving the sensing limits have been a major focus. Researchers have attempted to increase the sensors' sensitivities by optimizing the structures of the sensing fibers, with variations such as D-type, U-shaped, S-shaped and tapered fibers and the tapered sensing fiber is a novel sensing structure [4–7]. Tapered optical fiber sensors for label-free biomolecule detection have also been developed and evaluated with an immunoglobulin G antibody-antigen pair [8].

Besides, sensitive film decoration on optical fibers has also been introduced to improve the sensitivity [9–12]. Evanescent field optical fiber sensors of high sensitivity are obtained by embellishing the surface of the sensing fibers with biochemical reagents or films sensitive to the analyte. A nano-taper optical fiber coated with an ultrathin palladium film is used to detect low concentrations of hydrogen with fast response time [13]. In addition, with the recent remarkable progress in surface plasmon resonance (SPR) and photonic crystal fiber (PCF) technologies, micro- and nano-structure modified optical fiber sensors have attracted growing attention [14–18]. Gold thin film modification on optical fiber biosensors based on surface plasmon resonance has been proposed to enhance the SPR effect on biosensing [19,20]. Moreover, SPR phenomena are also found in noble metal nanoparticles, namely the localized surface plasmon resonance (LSPR).

Researchers have combined the advantages of optical fiber sensors and the performance of LSPR to improve the sensitivity [21,22]. Noble metal nanoparticles are decorated on the surface on the sensing fiber, and a localized surface plasmon resonance occurs which is excited by the evanescent wave around the bare core. A novel class of fiber-optic evanescent-wave sensor is constructed on the basis of modification of the unclad portion of an optical fiber with self-assembled gold colloids and the colloidal gold surface is functionalized with biotin, with a detection limit for streptavidin of 9.8 × 10^−11^ M [23]. To achieve stable test results, most commercial LSPR sensors use gold for the metal nanoparticles, but for the LSPR effect, silver nanoparticles are better [24]. In this paper, we provide a complete explanation of the sensor fabrication and propose a MEMS microchip in addition to describing the sensing fiber decoration in detail. Meanwhile, previous researchers always talk about the local surface plasma resonance peak wavelength variation due to the concentration of the analyte, but here we mainly focus on the intensity changes caused by the analyte absorbance, which is found to be more sensitive. There are still some unexplained details such as what is the specific difference effect of the modification and how much is the sensitivity really improved for testing the same analyte. In this work, the evanescent field optical fiber sensor with silver nanoparticle modification has been successfully fabricated and experiments performed with different concentrations of analytes. Comparison of the same sensing fiber without any decoration and modified with silver nanoparticles for the detection of methylene blue solutions has also been presented.

## 2. Theory

**Figure 1.**
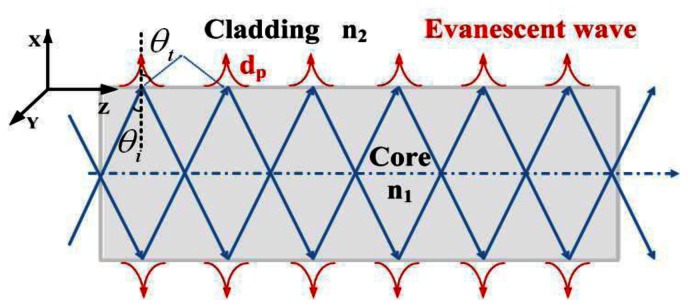
Schematic representation of the light path in optical fibers.

Optical fibers transmit light on the basis of the principle of total internal reflection (TIR) as shown in Figure 1.

When light propagates from the core to a cladding with low refractive index *n*_2_ and the incident angle is larger than the critical angle (*θ_i_* ≥ *θ_c_*), total reflection occurs. For the evanescent field optical sensor, the cladding is always peeled off and substituted by the absorptive analyte. Then the refractive index of the cladding region should be described as *n*_2_ = *n*_2_*_r_* + *jn*_2_*_j_*, where the real part shows the transmission characteristics of the refractive index, and the imaginary part represents the absorption properties. The propagation constant can also be written as *β* = *β*_2_*_r_* + *jβ*_2_*_j_*. In order to simplify the calculation process, here we consider the propagating wave near the refractive point to be the plane wave. The electric field is:

(1) $$E_{2}(x)=E_{20}\exp(jk_{0}n_{2}x\cos\theta_{t})\exp[j(\beta z-\omega t)]$$

According to the Snell's law, *n*_1_sin*θ_i_* = *n*_2_sin*θ_t_*, then $\sin\theta_{i}\geq \left|\overset{n_{2}}{\;}/\underset{n_{1}}{\;}\right|$, so Equation (2) can be obtained:

(2) $$\cos\theta_{t}=\pm j\sqrt{\frac{{n_{1}}^{2}}{{n_{2}}^{2}}\sin^{2}\theta_{i}-1}$$

Combining Equation (2) into Equation (1), we can get:

(3) $$\begin{array}{l} E_{2}(x)=E_{20}\exp\left[-k_{0}n_{2r}x\sqrt{\frac{{n_{1}}^{2}}{{n_{2}}^{2}}\sin^{2}\theta_{i}-1}\right]•\exp\left[-jk_{0}n_{2j}x\sqrt{\frac{{n_{1}}^{2}}{{n_{2}}^{2}}\sin^{2}\theta_{i}-1}\right]\\ •\exp(-\beta_{j}z)•\exp[j(\beta_{r}z-\omega t)] \end{array}$$

As shown in Equation (3), the evanescent field decays exponentially in the cladding (x-direction). When the evanescent field decays to 1/e of its value at the core-cladding interface, we can get the penetration depth of the evanescent field:

(4) $$d_{p}=\frac{\lambda }{2\pi n_{1}•\overset{\left|n_{2r}\right|}{\;}/\underset{n_{2}}{\;}\sqrt{\sin^{2}\theta_{i}-\frac{{n_{2}}^{2}}{{n_{1}}^{2}}}}$$

Usually, *n*_2_*_r_* ≪ *n*_2_, then the penetration depth is mathematically described as:

(5) $$d_{p}=\frac{\lambda }{2\pi n_{1}\sqrt{\sin^{2}\theta_{i}-\frac{{n_{2}}^{2}}{{n_{1}}^{2}}}}$$

where *λ* is the wavelength of the light source. As the wavelength becomes larger, the penetration depth increases.

## 3. Sensing Segment Fabrication

### 3.1. MEMS Microfluidic Channel Chip Fabrication

Considering the sensor fabrication process, a cladding segment (2 cm long) of the optical fiber was totally removed to make it the sensing fiber. The hydrofluoric acid wet etch method was adopted in the experiments. The required diameter of the sensing fiber was less than 8 μm, which makes it very thin and fragile, hence a microfluidic channel chip based on the micro electromechanical systems (MEMS) technology is presented, which contains a main deep channel in the middle and two microfluidic channels branches in the two sides as inlets and outlets of the analyte solution.

**Figure 2.**
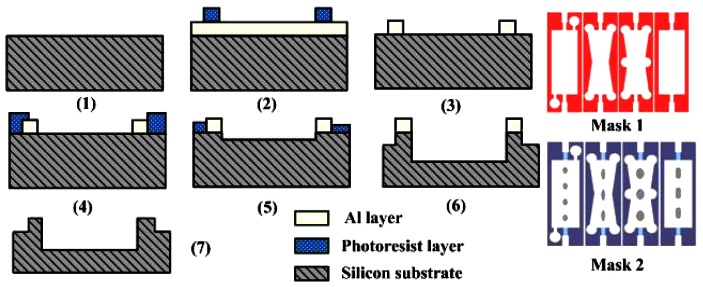
Fabrication process sequence and the mask pattern.

**Figure 3.**
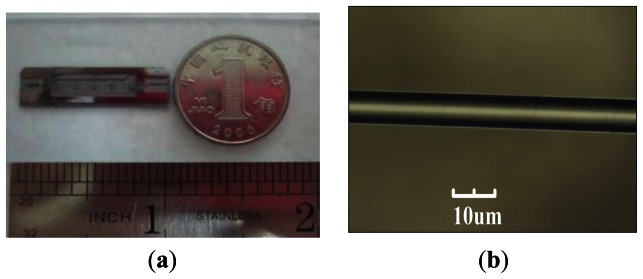
(**a**) Front-side photograph of the MEMS microchannel chip; (**b**) Completed sensing fiber picture.

Figure 2 illustrates the microchannel chip process based on MEMS technology. The fabrication process is rather simple: (1) a clean (100) n-type single side polished silicon wafer with 380 um thickness was prepared; (2) a 1 μm thick aluminum layer was deposited by DC magnetron sputter and then a 2 μm photoresist layer was deposited on it; one side of the silicon wafer lithography was completed utilizing Mask 1, which transferred the pattern onto the photoresist layer; (3) the aluminum layer was patterned by the IBE ion beam etch method; (4) another photoresist layer is deposited and then lithography was done using Mask 2; (5) the photoresist layer was patterned by an inductively coupled plasma ICP process while the aluminum layer was etched about 50 μm; (6) the photoresist layer was totally removed, then the aluminum layer was still etched by ICP of 200 μm thickness. (7) the residual aluminum layer was removed by the wet etching method and the microchannel chip was successfully completed, as shown in Figure 3(a).

The cladding etching solution contained 1:1:1 hydrofluoric acid (HF), acetic acid and deionized water. The experiential etching rate was 20 μm/h within the cladding region and when etched to the core, the reaction rate sped up considerably. Figure 3(b) shows the completed sensing fiber with a diameter of about 8 μm.

### 3.2. Optical Sensing Fiber Decorated with Silver Nanoparticles

#### 3.2.1. Preparation of Ag Nanoparticles

Polyol synthesis was originally developed by Fievet and colleagues as a simple and versatile route to colloidal particles made of metals and alloys, with typical examples including Ag, Au, Cu, Co, Ir, Ni, Pd, Pt, Ru, CoNi, and FeNi [25]. In a typical synthesis of silver nanospheres, anhydrous ethylene glycol (5 mL, 99.8%, Aldrich) was heated at 160 °C for 1 h; 3 mL of ethylene glycol solution of AgNO3 (0.25 M, 99+%, Aldrich) and 3 mL of ethylene glycol solution of polyvinyl pyrrolidone, PVP (2.5 M, weight-average molecular weight ≈ 55,000, Aldrich) were simultaneously injected into the reaction flask over 10 minutes; then the reaction mixture was heated at 160 °C for another 45 minutes.

**Figure 4.**
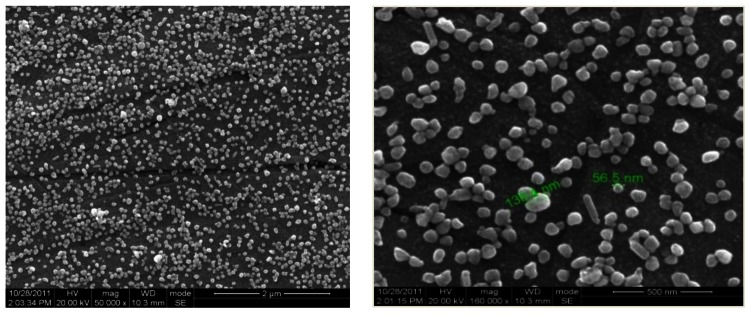
The SEM images of the as-prepared silver nanoparticles.

Figure 4 shows the SEM image of the silver nanospheres that were prepared when the ratio between PVP and AgNO_3_ in the reaction mixture was 10. These silver nanospheres' average diameter is 85 nm and the smallest is 56.5 nm. These particles are essentially spherical in shape although a few slightly anisotropic particles can be observed. The formation of anisotropic colloids might be ascribed to preferential addition of silver atoms at the high-energy twin sites, resulting in the formation of relatively short rods, or to the geometric limitations imposed by the faceted nature of the silver crystal.

#### 3.2.2. Ag-Modified Optical Fibers

The Ag-modification on the sensitive segment of the sensor fiber is an important part of the experiments. In order to preferably fix the metal particles to the surface of the sensing optical fiber, some functionalization treatments need to be performed.

**Figure 5.**
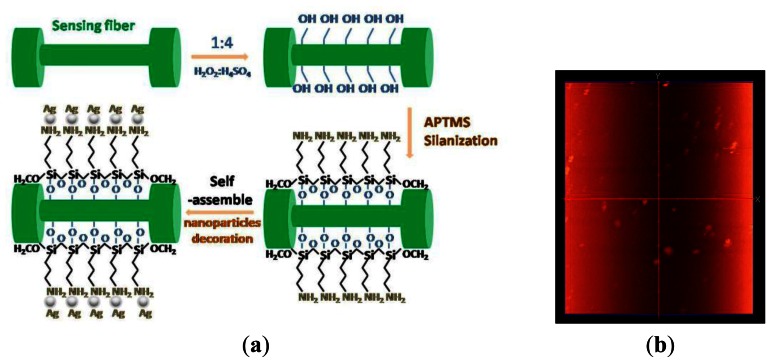
(**a**) Schematic of sensing fiber surface silanization and the silver nanoparticles modification processing procedure; (**b**) Image of the sensing fiber modified with the silver nanoparticles.

The specific processing steps were as follows: firstly, the unclad portion (∼2 cm) of the optical fibers was cleaned for 30 min in a bath consisting of one part concentrated nitric acid (HNO_3_) to three parts concentrated hydrochloric acid (HCl). Secondly, after thoroughly rinsing withy de-ionized water, the sensing part is submerged in piranha solution (1:4, 30% H_2_O_2_/H_2_SO_4_) at 80 °C, sonicated for 30 min and sufficiently cleaned with de-ionized water. The piraha solution here could remove the retained plastic cladding and supply hydroxyl groups to the sensing surface. Thirdly, the clean unclad portion of the optical fibers was then immersed into a 1:10 solution of 3-aminopropyltrimethoxysilane (APTMS) silane coupling agent and methanol for 24 h, which formed amino groups on the surface. Here, APTMS works as a surface modification linker, which provides amino groups with positive electric charge on the sensing fiber, then the Ag nanoparticles with negative electric charge can self-assemble on the surface of the sensing fiber because of the electrostatic force produced [17]. Finally, the clean unclad sensing section is dipped into the prepared silver nanoparticles solution for 24 h. Subsequently, the modified optical fibers are repeatedly rinsed with de-ionized water and dried under N_2_. Figure 5(a) illustrates the principle of surface silanization and modification with silver nanoparticles, and Figure 5(b) shows the size and distribution of Ag nanoparticles decorated on the sensing fiber.

## 4. Experiments and Discussion

### 4.1. Experimental System

**Figure 6.**
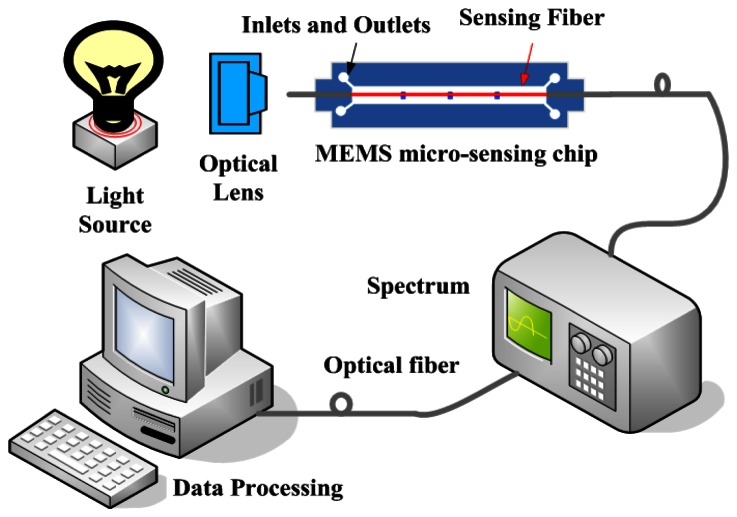
Schematic representation of the experimental system.

The experimental setup system employed to detect different analytes and the optical response of the sensor is shown in Figure 6. The setup consists of the light source, the sensing section with a sensing fiber of a 2 cm long unclad portion and a MEMS micro-sensing chip, a scientific-grade UV-Vis spectrometer (USB4000, Ocean Optics Inc., Dunedin, FL, USA) and a data processing computer. The silver nanoparticle-decorated sensing fiber was fixed in the prepared MEMS sensing chip. The transmitted light was then collected and measured by spectrometer calculating the intensity of light toward the wavelength with associated data processing software (SpectraSuite, Ocean Optics inc., Dunedin, FL, USA). The absorption medium used for experiments is methylene blue dissolved in deionized water at different concentrations. Methylene blue is usually used for biological, bacteria tissue staining, and the detecting of methylene blue is often applied in biological marking and identification. It can be effectively absorbed on the polar sensing fiber surface with OH^−^ions due to its chemical properties and the absorption peak of methylene blue is around 600 nm.

### 4.2. Result and Discussion

**Figure 7.**
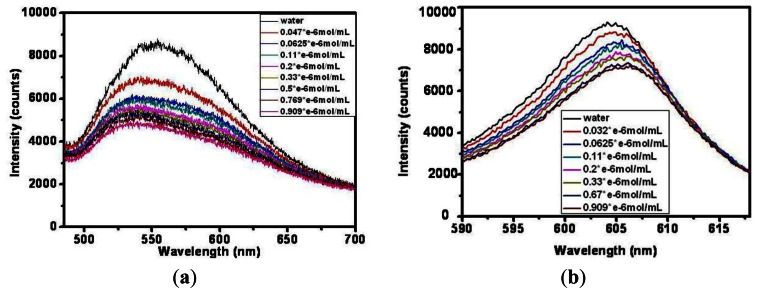
Spectra of varying concentrations of methylene blue under (**a**) visible light; (**b**) red light.

Firstly, an evanescent field optical sensor without any decoration is successfully fabricated with a 2 cm unclad sensing segment. The incident wavelength is 500–700 nm, and different concentrations of methylene blue solutions ranging from 0 to 0.909 μmol/mL are detected as shown in Figure 7(a). As the concentration increases, the absorption of the analyte by the evanescent field around the sensing fiber becomes large and the output intensity gradually deceases. The spectra of the nine concentrations can just be distinguished from each other under this light source. For better analysis, this same experiment has also been carried out under a narrow incident light band. Figure 7(b) illustrates the spectra of varying concentrations of methylene blue under a red LED light. The evanescent field reacts mightily with the analyte because the wavelength grows from 520–550 nm to 600–610 nm, which is in accordance with Equation (5) that shows that as the wavelength increases the penetration depth becomes large.

**Figure 8.**
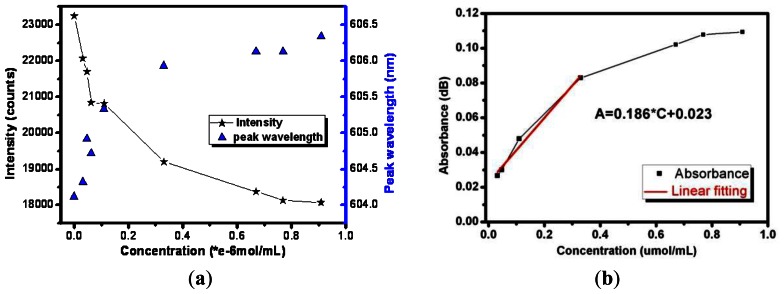
(**a**) The curves of the peak absorption intensity and wavelength changing with different concentrations of the analytes; (**b**) The plot of absorbance *versus* concentration.

Besides, the curves of the peak absorption intensity and wavelength changes with the concentration of the analytes are also obtained (Figure 8(a)). With the increase of the concentrations of analyte, the absorption peak intensity decreases, accompanied by a minor red shift of the peak wavelength. When the concentration grows larger than 0.4 μmol/mL, the absorption peak intensity changes slowly and peak wavelength red shift becomes inconspicuous. The sensor could not distinguish the changes in light intensity if the concentration continues to rise.

By calculating the ratio of power before and after each modification method, the absorbance value can be obtained and then specific values of sensitivity can also be obtained. After conducting the experiments, by obtaining the difference of the absorbance values Δ*A*, which is obtained by detecting different concentrations of the analytes, and the increment of the concentrations, we can calculate the actual sensitivity of the sensor, which is *M*′ = Δ*A*/Δ*C*, where, the absorbance can be calculated by $A=-\log_{10}\left[\frac{P_{\text{out}}}{P_{\text{in}}}\right]$.

According to the experimental sensitivity definition, the absorbance of corresponding concentration is obtained and a plot of absorbance *versus* concentration is depicted in Figure 8(b). The linear sensing region of the sensor is 0–0.4 μmol/mL with linearity of 0.977. Hence, in the linear range, the sensitivity for methylene detection of the evanescent field optical fiber without decoration is 0.186, that is, when the concentration changes by 0.1 μmol/mL it can cause the absorbance to vary by 0.186 dB.

**Figure 9.**
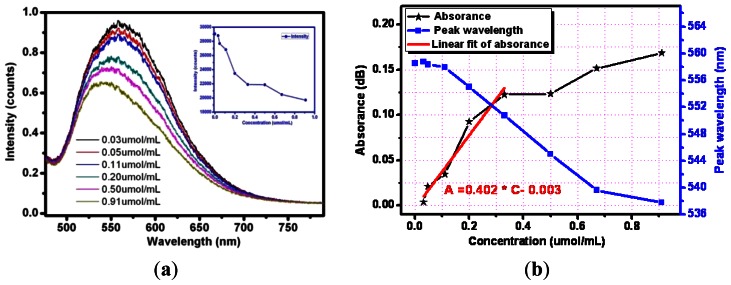
(**a**) Spectra of six different concentrations of methylene blue; (**b**) Test curve of absorbance *versus* corresponding concentration and plot of absorbance *versus* concentration of the silver nanoparticle-modified optical fiber sensor.

Next the optical responses and properties of silver nanoparticle-modified optical fiber sensor were tested and analyzed. Silver nanoparticles were decorated on the sensing surface by the method mentioned above. Experiments using the prepared sensing fiber to detect different concentrations of methylene blue solutions under visible light was performed. Figure 9(a) gives examples of the response of silver nanoparticles fixed on sensing fiber surface containing exposed to increasing concentrations of methylene blue solutions (from top to bottom). For the lower concentration analyte the output intensity seems to be higher after being absorbed by the evanescent field. As the concentration varied from 0.03 μmol/mL to 0.9 μmol/mL, the output light intensity weakens gradually.

The experimental test curve of absorbance *versus* corresponding concentration is described in Figure 9(b). Over the range of the methylene blue concentrations from 0 to 0.4 μmol/mL, the sensor response is linear (R = 0.9496). An analyte variation of 0.1 μmol/mL can cause an absorbance change of 0.402 dB, so the Ag nanoparticle-decorated optical fiber sensor's sensitivity in this region is 0.402.

Moreover, the evanescent wave around the sensing fiber excites the resonance of the silver nanoparticles and a resonance peak wavelength shift is also observed. A minor concentration of the analyte increases the resonance peak wavelength shifts blue slightly. Analytes of changing concentrations have different refractive indices. For the methylene blue solutions with concentrations of 0.11 μmol/mL, 0.33 μmol/mL, 0.67 θmol/mL, the resonance peak wavelengths are 557.9 nm, 550.7 nm and 539.6 nm, respectively. The variation of the resonance wavelength as a function of the concentration is represented by the blue curve of Figure 9(b) and fits perfectly with a linear approximation with a correlation coefficient of 0.983. Meanwhile, comparison of the same sensing fiber without any decoration and modified with silver nanoparticles can also be made. The optical responses show that the output intensity of the Ag nanoparticle-modified sensor is enhanced as the evanescent wave excites the local surface plasma resonance. The sensitivity of the silver nanoparticle- modified evanescent field optical fiber sensor is higher for the methylene blue solution. The previous researchers always focused on the relation between the local surface plasma resonance peak wavelength variation and the concentration of the analyte [19,23], but the absorbance spectra are found to be more sensitive to the concentrations of methylene blue solution compared to the peak wavelength.

**Figure 10.**
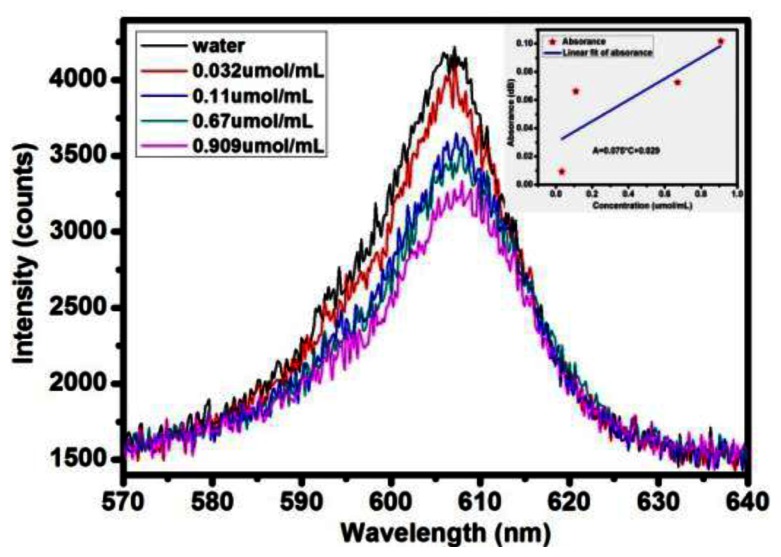
The test spectra of melamine.

Besides, we have tested different concentrations of melamine, which is dangerous and harmful to human health. Figure 10 shows the testing spectra of melamine using the silver nanoparticle-decorated sensing fiber, and the sensitivity of the detection result is 0.075. When the melamine concentration changes by 0.1 μmol/mL the absorbance changes by 0.075 dB. Overall, the evanescent field optical sensor can potentially provide rapid, real-time toxic sample detection in biological and chemical applications.

## 5. Conclusions

Different concentrations of methylene blue solutions changing from 0 to 0.909 μmol/mL are detected by a Ag nanoparticle-modified evanescent field optical fiber sensor. Comparison of the same sensing fiber without any decoration and the silver nanoparticle-modified version is also presented. The sensitivity of the modified optical fiber sensor for the methylene blue solution detection is 0.402 and it is 0.186 for the sensing fiber without any decoration. The absorbance spectra are found to be more sensitive to the concentrations compared to the peak wavelength, which is different from the previous research focus. Besides, a microchannel chip based on the Micro Electromechanical Systems (MEMS) technology is presented to produce an unclad sensing fiber section of high quality. The evanescent field optical sensor is low cost, highly sensitive and convenient, and can potentially operate for rapid, real-time and continuous detection in applications without the need for labeling or fluorescence of the biochemical analyte of interest.
